# Variations of Symptoms from the Type

**Published:** 1890-02-01

**Authors:** 


### VARIATION OF SYMPTOMS FROM THE TYPE.

We have on several occasions referred to the variation of symptoms of disease, remarking that scarcely any two cases of the same malady present a typical portrait. Many cases of the same disease are, in fact, merely a blurred image of the portrait. Often one particular symptom is the prominent one, and often a particular symptom which figures foremost in the portrait is slight, obscured, or even absent altogether. We have recently noticed several apt illustrations of the above. Dr. O. H. Merril, of Corinna, gives in the New York *Medical Record* of December 14th a case of remarkable hæmorrhage in typhoid fever, where "the bowel symptoms were conspicuous by their absence." But "without any warning whatever there was a perfectly overwhelming discharge of blood per rectum," bringing the patient to an almost moribund condition. But he eventually recovered, remaining well for some weeks, when alarming epistaxis occurred. This being stopped by plugging the anterior and posterior nares, blood began to drop from one eye at the rate of thirty drops per minute. Blood was also discharged from the penis and mouth. Eventually the patient recovered. Now, it is probable, although Dr. Merril does not say so, that the patient had some hereditary tendency to hæmophilia, which developed during the attack of typhoid. In the same journal Dr. Joseph M. Study, of Cambridge City, Ind., records two cases of high temperature in typhoid fever. In the one case, for twenty-six days the recorded temperature was from 103½deg. to 105½deg. F., the average being 104¼deg. In the other case, for thirty-two consecutive days the temperature was 103½deg. to 106⅗deg., the average being 104⅘deg. Dr. Study observes that many persons die from typhoid with a very moderate increase of temperature, and it cannot be denied that there are individuals who possess an idiosyncrasy to resist the high temperature of diseases. Also a higher temperature than usual may be referred to some unexplainable idiosyncrasy or peculiarity of constitution. It is not, however, in typhoid fever only that great variation of symptoms are encountered. There is scarcely any malady more variable in its effects or symptoms than cholera. The influenza epidemic also affords an illustration, chest affection predominating in some cases, while muscular pains are most prominent in others. The accounts of diseases we read in text-books are very different to what we meet with in actual practice, and, as a rule, authors do not sufficiently dwell on variability of symptoms.

